# University of the State of New York.—Examinations Department

**Published:** 1891-07

**Authors:** 


### UNIVERSITY OF THE STATE OF NEW YORK.—EXAMINATIONS DEPARTMENT.

The following amendment to Chapter 507 Laws of New York, 1890, was approved by the Governor on May 4th, taking effect immediately, as Chapter 311 of the Laws of 1891:

This act shall not apply to any student who duly matriculated in some legally incorporated medical college of the State of New York before the fifth day of June, 1890, provided that such student, within three months after the enactment of this amendment, shall file with the Secretary of the Board of Regents of the University of the State of New York, a certificate setting forth the fact of such matriculation, verified by the applicant and signed by the Secretary of the Faculty of the college at which he matriculated.

This act will exempt only such students as file the required certificate in the Regents' office on or before August 4, 1891.

---

CERTIFICATE.

*It is hereby certified* that was on 18 duly registered on its official records as a fully matriculated medical student in

(Signed) *Secretary of the Faculty.*

State of New York
City of } ss.
County of being duly sworn, says that he is the identical person referred to in the above certificate, and that all the statements therein set forth are true.

Sworn to before me this day of 1891. } ..........................................

*Notary Public.*

---

The general addresses before the American Medical Association, at its Washington meeting in May, were of more than usual merit, and will repay careful reading.

Dr. E. L. Shurly, of Detroit, delivered the address on Medicine, choosing for his subject, The Relations of Microörganisms and Toxines to the So-called Zymotic or Infectious Diseases. It is replete with science, and will prove of great interest to general practitioners as well as specialists. It is published in full in the Journal of the Association for May 23, 1891. The address on State Medicine, by W. L. Schenck, M. D., of Topeka, Kans., will also be found in the same number, and will likewise prove profitable reading. The address on Surgery fell to the lot of Dr. Joseph M. Mathews, of Louisville, and it is prudent to assert that the selection was fortunate. Dr. Mathews is one of the most scholarly men in the profession, and combines profundity with eloquence in a most happy manner—joint accomplishments that but few possess. His subject was, Stricture of the Rectum — Its Etiology, Pathology, Symptomology, Diagnosis, and Treatment. If the surgeon desires the latest thought on this important subject, presented in a most attractive form he will surely read this paper in the Association Journal for May 9, 1891.

The appointments for 1892 are: Address on General Medicine —Dr. J. S. Cain, of Tennessee; Address on General Surgery—Dr. John B. Hamilton, of Illinois; Address on State Medicine—Dr. C. A. Lindsly, of Connecticut.

